# The risk of QTc-interval prolongation in COVID-19 patients treated with chloroquine

**DOI:** 10.1007/s12471-020-01462-6

**Published:** 2020-07-09

**Authors:** F. S. Sinkeler, F. A. Berger, H. J. Muntinga, M. M. P. M. Jansen

**Affiliations:** 1 Department of Clinical Pharmacy, Elisabeth-TweeSteden Hospital, Tilburg, The Netherlands; 2 Department of Cardiology, Elisabeth-TweeSteden Hospital, Tilburg, The Netherlands; 3 Department of Clinical Pharmacy, Meander Medical Centre, Amersfoort, The Netherlands

**Keywords:** Chloroquine, COVID-19, Electrocardiography, QTc interval

## Abstract

**Background:**

Chloroquine, a quinolone antimalarial drug, is known to potentially inhibit pH-dependent viral replication of the SARS-CoV‑2 infection. Therefore, chloroquine is considered as a treatment option for coronavirus disease 2019 (COVID-19). Chloroquine is known for prolonging the QT interval, but limited data are available on the extent of this QT-prolonging effect.

**Objective:**

To assess the QTc-prolonging potential of chloroquine in COVID-19 patients and to evaluate whether this prolongation increases with the cumulative dose of chloroquine and is associated with the peak plasma concentration of chloroquine. Furthermore, the number of patients who prematurely discontinued treatment or had an adjustment in dose due to QTc-interval prolongation was established.

**Methods:**

A retrospective, observational study was performed in patients aged over 18 years, hospitalised for a suspected or proven infection with COVID-19, and therefore treated with chloroquine, with a baseline electrocardiogram (ECG) performed prior to the start of treatment and at least one ECG after starting the treatment.

**Results:**

In total, 397 patients were included. The mean increase in QTc interval throughout the treatment with chloroquine was 33 ms. Nineteen out of 344 patients unnecessarily had their treatment prematurely discontinued or adjusted due to a prolonged QTc interval based on the computerised interpretation of the ECG.

**Conclusion:**

Chloroquine treatment in COVID-19 patients gradually increased the QTc interval. Due to a significant number of overestimated QTc intervals by computer analysis, it is advisable to measure the QTc interval manually before adjusting the dose or withdrawing this potentially beneficial medication.

## What’s new?

- Chloroquine treatment increases the QTc interval.
- The timing of the electrocardiogram recording within the dosing interval is not relevant.
- Within the treatment period of 5 days, the QTc interval continues to increase.
- It is advisable to monitor QTc intervals throughout the treatment period.
- It is advisable to measure the QTc interval manually before adjusting the dose or withdrawing the treatment.

## Introduction

Chloroquine, a quinolone antimalarial drug, is known to inhibit pH-dependent viral replication in vitro for severe acute respiratory syndrome coronavirus (SARS-CoV-1) and several other viruses [1, 2]. Chloroquine can also inhibit the viral replication of SARS-CoV‑2 as the necessary concentration (EC50) can be reached with a cumulative dose of 3300 mg [3, 4]. Chloroquine has been considered as a treatment option in the Dutch guidelines since the beginning of the coronavirus disease 2019 (COVID-19) outbreak in the Netherlands [5].

A common side effect of chloroquine is prolongation of the QT interval. It is on the CredibleMeds list of drugs associated with a ‘known risk of torsades de pointes (TdP)’ [6]. Limited data on the extent of this QT-prolonging effect are available from trials where chloroquine was used as an antimalarial drug [7, 8]. Furthermore, the dosages used in these trials for malaria were lower and the duration was shorter than in the therapy for COVID-19. Recently, a study with 95 patients treated with chloroquine for COVID-19 was published [9]. This study found a mean increase in the QTc interval of 35 ms, which is remarkably longer than the previously described prolongations of 6 and 16 ms [7, 8].

In order to further evaluate the QTc-prolongation potential of chloroquine, we conducted a retrospective observational study. The main aim of this study was to assess the QTc-prolongation potential of chloroquine in COVID-19 patients.

## Methods

This retrospective, observational cohort study was conducted at two teaching hospitals in the Netherlands (Elisabeth-TweeSteden Hospital (ETH) in Tilburg and Meander Medical Centre (MMC) in Amersfoort) from 10 March until 22 April 2020. All patients aged over 18 years, hospitalised for a suspected or proven infection with COVID-19, and therefore treated with chloroquine, with a baseline electrocardiogram (ECG) performed prior to the start of treatment and at least one ECG after starting the treatment with chloroquine were included. Due to the retrospective nature of this study, the medical ethical committee of Brabant waived the requirement for individual informed consent.

The main outcome measure was the difference in QTc time (∆QTc) between the QTc interval of the baseline ECG (ECG-0) and the first ECG taken after the start of the chloroquine treatment (ECG-1). Secondary outcome measures were the ∆QTc between the QTc interval of ECG‑0 and the last available ECG during chloroquine treatment (ECG-L), and whether the timing of the ECG during the second dosing interval of chloroquine had a relevant effect on the ∆QTc found as the primary outcome measure. We studied the association of several known risk factors associated with an increase of the QTc interval. For patients from the ETH population, where chloroquine treatment was stopped because of a prolonged QTc interval (>500 ms, or an increase >60 ms from baseline), the QTc interval was manually recalculated by a cardiologist to verify the justification for stopping chloroquine treatment.

All patients admitted to the hospital with a suspected or proven infection with COVID-19 were treated with chloroquine according to the Dutch guidelines [5]. The dosing regimen for chloroquine consisted of a loading dose of 600 mg followed by 300 mg twice daily, starting 12 h after the loading dose. The duration of the total regimen was 5 days, reaching a cumulative dose of chloroquine of 3300 mg. The exact administration date and time for all the chloroquine administrations were extracted from the electronic patient record.

The following patient characteristics were obtained from the medical record: sex, age, weight and body mass index, renal function at the start of chloroquine treatment, electrolyte levels prior to and during treatment (potassium, magnesium and calcium) and duration of chloroquine treatment. Comorbidity at the start of the treatment was classified by the Charlson Comorbidity Index. Relevant concurrent use of other potentially QTc-prolonging co-medication, defined as medication with a ‘known risk of TdP’ according to the CredibleMeds list, was defined as at least one administration 24 h prior to or 48 h after the first dose of chloroquine [6]. The same was done for lopinavir-ritonavir since it is known to significantly increase chloroquine plasma concentration and it was initially mentioned as a potential treatment option for COVID-19 in the first version of the Dutch guidelines and was therefore used in combination with chloroquine [10].

A baseline ECG, including heart rate, PR interval, corrected QT interval and QRS duration, was performed prior to initiation of the therapy with chloroquine. The computerised values were used for interpretation using the Marquette 12SL ECG analysis programme (GE Healthcare, Chicago, IL, USA).

The baseline ECG had to be conducted within 1 month before the start of chloroquine therapy. During the COVID-19 pandemic, the first ECG after the start was preferably recorded 24–72 h after the initiation of the treatment. For this study, all the available ECGs recorded during the treatment period with chloroquine were extracted from the hospital information system Epic Systems Corporation (Madison, WI, USA) at the ETH, and from Easycare (Healthcare B.V., Deventer, The Netherlands) at the MMC. Available ECGs were allocated to the dosing interval in which they were recorded. Furthermore, the obtained calcium, potassium and magnesium levels within 12 h prior to or after recording of an ECG were linked to that ECG.

For patients in the ETH population who prematurely discontinued treatment or had a dose adjustment, and had a QTc interval above 500 ms and/or an increase of more than 60 ms from baseline, as measured on ECG during the latest dosing interval, the medical records were searched for the reason for premature discontinuation or adjustment in therapy. All ECGs from patients with an adjusted dose or discontinuation of therapy with a QTc interval >500 ms and/or an increase in QTc interval >60 ms were manually recalculated by a cardiologist using the method described by Postema and Wilde [11].

Data were analysed using IBM SPSS Statistics version 24.0 (Armonk, New York, NY, USA). Descriptive statistics were used to describe baseline characteristics. A linear regression analysis was performed to explore whether the timing of the ECG during the second dosing interval of chloroquine had a relevant effect on the ∆QTc found as the primary outcome measure. An independent *t*-test was used to determine whether there was a statistically significant difference in QTc prolongation for sex, renal function or potential QTc-prolonging co-medication. Potential QTc-prolonging co-medication was dichotomised as either use or no use of potential QTc-prolonging co-medication. Renal function was dichotomised as either a renal function above or under 60 ml/min per 1.73 m^2^. Simple linear regression analysis was used to predict QTc prolongation from age, baseline electrolyte levels or electrolyte levels around the first ECG after the start. Factors were considered statistically significant if *p* < 0.05. Factors that were associated with a probability of *p* < 0.05 in the univariate analysis were entered into multivariate models to adjust for confounding.

## Results

A total of 397 patients were included; 344 patients at the ETH and 53 patients at the MMC. These patients had a baseline ECG before starting treatment and at least one ECG during treatment. Baseline characteristics are displayed in Tab. 1.

**Table 1:** Baseline patient characteristics

Patient characteristics (*n* = 397)	Mean ± standard deviation
Age (years)	67.8 ± 12.5
Male^a^	262 (66%)
BMI (kg/m^2^)	28.5 ± 5.6
eGFR <60 (ml/min per 1.73 m^2^)^a^	116 (42%)
Use of potential QTc-prolonging co-medication^a^	106 (27%)
Concurrent use of antiarrhythmic drugs^a^	10 (3%)
Comorbidities^a^	
– Myocardial infarction	38 (10%)
– Congestive heart failure	32 (8%)
Electrolyte levels prior to starting treatment	
– Potassium (mmol/l)	4.2 ± 0.5
– Calcium (mmol/l)	2.2 ± 0.2
– Magnesium (mmol/l)	0.8 ± 0.1

^a^Sex, eGFR, potential QTc-prolonging co-medication, concurrent use of antiarrhythmic drugs and comorbidities are presented as numbers (%)

*BMI* body mass index, *eGFR* estimated glomerular filtration rate using Chronic Kidney Disease Epidemiology Collaboration (CKD-EPI) formula

**Fig. 1:**
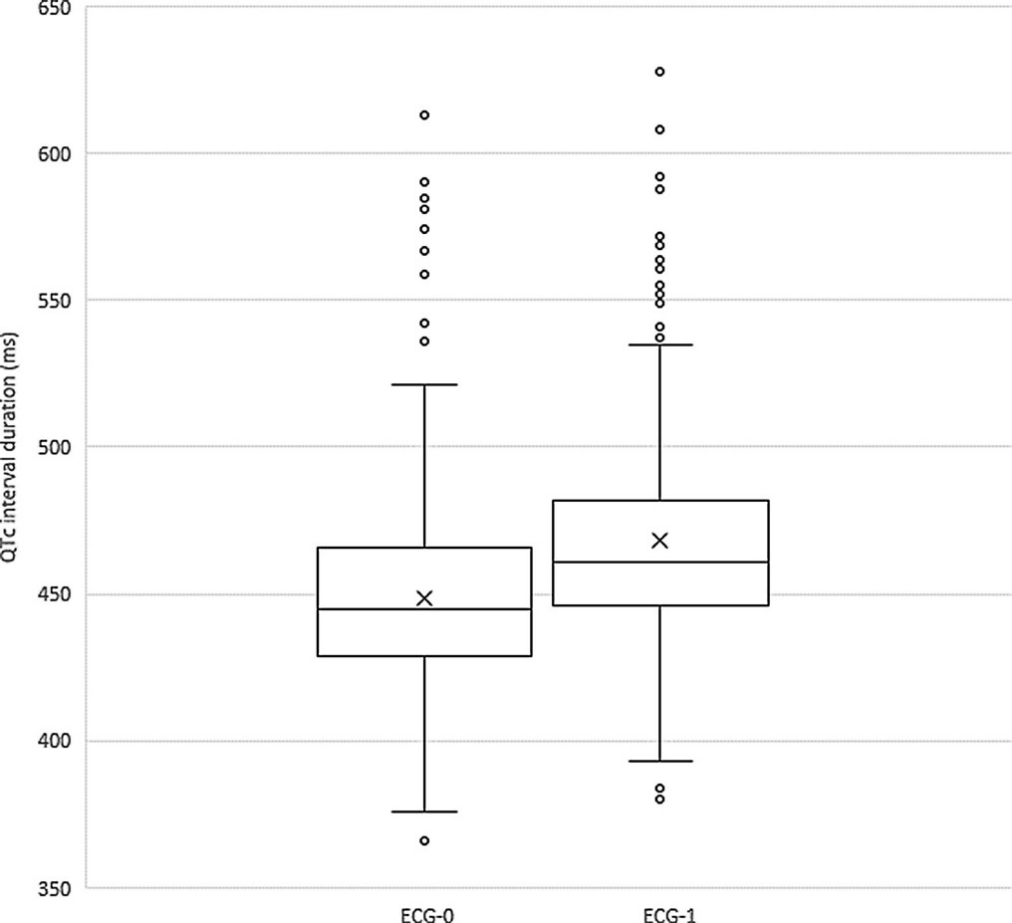
Median QTc interval with interquartile range for the baseline electrocardiogram (*ECG‑0*) and the first ECG after the start of treatment (*ECG‑1*), based on 397 patients. The mean QTc interval is displayed as *X*

Treatment with chloroquine resulted in a mean QTc prolongation [±standard deviation (SD)] of 20 ± 39 ms between ECG‑0 and ECG‑1. Using computerised interpretation, the mean QTc interval before treatment was 448 ± 34 ms, whereas the mean QTc interval of ECG‑1 was 468 ± 38 ms. This difference was statistically significant with *p* < 0.05. The corresponding QRS intervals were 98 ± 20 ms, 100 ± 22 ms and 101 ± 21 ms for ECG‑0, ECG‑1 and ECG‑L respectively. Fig. 1 shows the median, quartiles and mean QTc interval for ECG‑0 and ECG‑1.

**Fig. 2:**
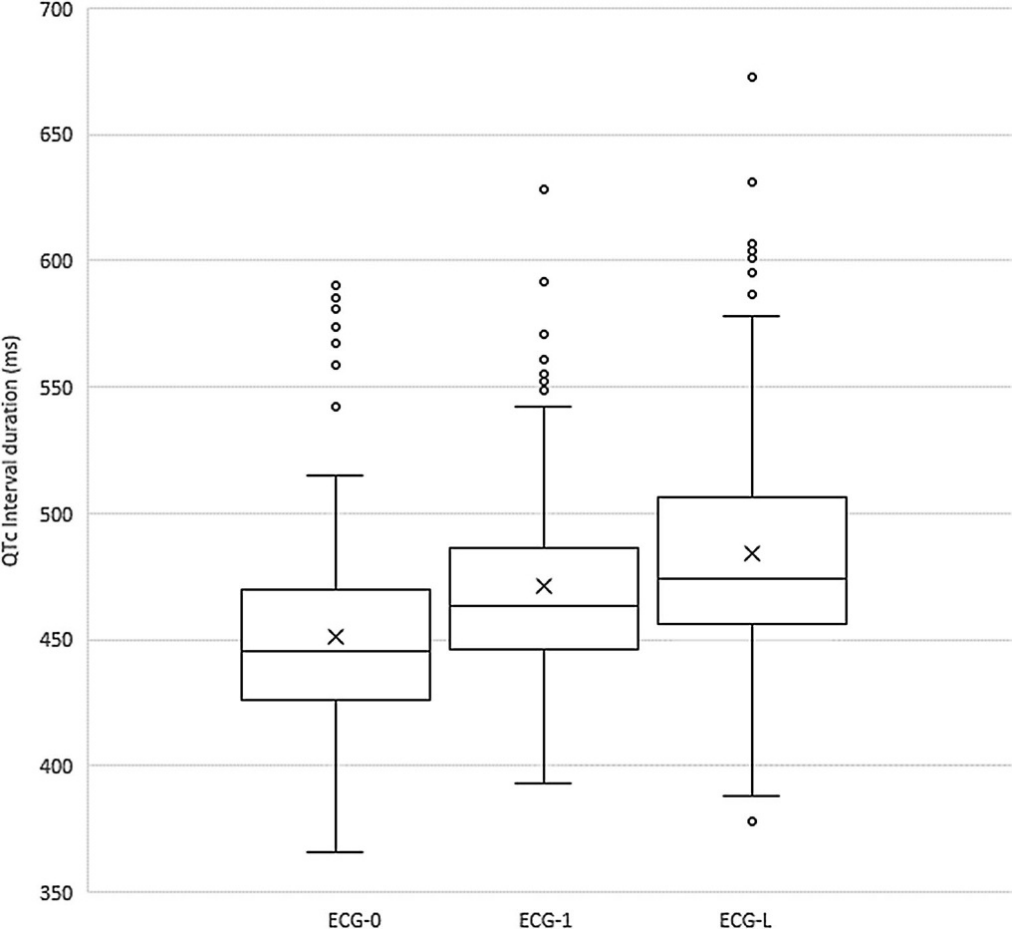
Median QTc interval with interquartile range for the baseline electrocardiogram (*ECG‑0*), the first ECG after the start of treatment (*ECG‑1*) and the last available ECG during chloroquine treatment (*ECG‑L*), based on 155 patients. The mean QTc interval is displayed as *X*

To evaluate the relationship between the QTc interval and the cumulative dose of chloroquine, ∆QTc between ECG‑0 and ECG‑L was evaluated. Intervals were defined as the time between two administrations of chloroquine, where interval 1 was the time between the loading dose of 600 mg and the following dose of 300 mg. In most patients, only a baseline ECG and one ECG after start were measured. However, 155 patients had more ECGs recorded during the treatment. In these patients, the mean dosing interval in which the first ECG after the start was recorded was 2 and the mean interval in which the latest ECG was recorded was 6. From these 155 patients, ∆QTc was calculated for ECG‑0 and ECG‑L. Fig. 2 displays the median, quartiles and mean QTc interval for ECG‑0, ECG‑1 and ECG‑L. For the 155 patients, the mean QTc prolongation between ECG‑0 and ECG‑1 was 20 ms (±43 ms). The mean QTc prolongation between ECG‑0 and ECG‑L was 33 ms (±53 ms). The differences in QTc interval for ECG‑0, ECG‑1 and ECG‑L were all statistically significant with a *p*-value of <0.05. In addition, linear regression analysis demonstrated a significant correlation between the increase in QTc interval and duration of treatment.

**Fig. 3:**
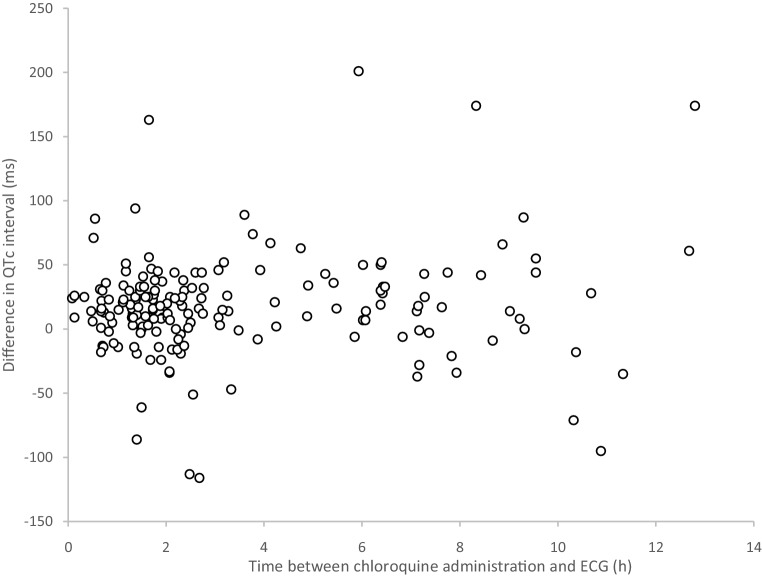
Difference between QTc from the baseline electrocardiogram (*ECG‑0*) and the QTc measured in dosing interval 2 (between second and third administration) plotted against the time between the administration of the second dose of chloroquine and measurement of the ECG

To evaluate whether the risk for QTc prolongation increased as a function of the plasma drug concentration (*C*_max_) during a dosing interval, QTc intervals measured at different time-points during interval 2 (between the second and third administration) were determined for the 179 patients who had an ECG performed in chloroquine dosing interval 2. Fig. 3 displays the time after the second administration of chloroquine and the difference between the baseline QTc and the QTc in dosing interval 2.

Sex and renal function were not significantly correlated with the ∆QTc between ECG‑0 and ECG‑1. Baseline electrolyte levels and those measured around ECG‑1 or age were not associated with the ∆QTc between ECG‑0 and ECG‑1. Only the use of potential QTc-prolonging co-medication had a statistically significant effect on QTc prolongation (24 ± 47 ms) between ECG‑0 and ECG‑1, compared to no use of QTc-prolonging co-medication (18 ± 36 ms), *p* = 0.004. However, this was not considered clinically relevant. Univariate analysis revealed only potential QTc-prolonging co-medication to be a risk factor and therefore a multivariate analysis was not performed.

Seventeen out of 397 patients had a baseline QTc interval exceeding 500 ms using computerised interpretation. After consulting a cardiologist, treatment with chloroquine was started in all patients. During treatment with chloroquine, 63 patients had a QTc interval exceeding 500 ms and/or had an increase in QTc >60 ms. Non-sustained ventricular tachycardia was observed in one patient, who had a manually calculated baseline QTc interval of 481 ms. After the third dose, the manually calculated QTc interval of this patient had increased to 540 ms.

Only the group of patients admitted to the ETH was used to identify the number of patients with premature discontinuation or with an adjustment in therapy due to QTc prolongation. Of the 344 patients, 50 patients (14.5%) had prematurely discontinued or had a dose adjustment of chloroquine. In 27 of these 50 patients (54%), chloroquine was discontinued and three patients had a dose reduction to 150 mg twice daily due to QTc prolongation. These clinical decisions seem to have been based on the computerised interpretation of the ECG. The ECGs of these 30 patients were manually recalculated by an independent cardiologist. Chloroquine treatment resulted in a mean prolongation of 75 ms for the computerised interpretation and 43 ms for the manually calculated QTc interval. The manual interpretation disclosed that only 11 patients indeed had a QTc interval of at least 500 ms and/or an increase in QTc of more than 60 ms.

## Discussion

Our study shows that treatment with chloroquine in COVID-19 patients significantly prolongs the QTc interval with a mean QTc prolongation of 33 ms throughout the treatment. QTc prolongation, defined as a QTc interval above 500 ms or an increase of more than 60 ms from baseline, was seen in a considerable number of patients (16%), even resulting in ventricular tachycardia in one patient.

The QTc interval seemed to increase continuously after initiation of therapy. This could possibly be explained by the apparent half-life of 1.6 days [12]. Due to this half-life, the plasma concentration will further increase during the 5 days of treatment and steady state would only be reached 7 days after starting therapy. This is supported by the concentration-time profile of chloroquine, where the cumulative dose of chloroquine is highest at the end of the treatment period [12]. Thus, QTc prolongation and the associated risk of TdP will continue to increase up until the end of the 5‑day treatment period.

A study in healthy volunteers showed the QTc prolongation to be greatest 4 h after the second dose of chloroquine [7]. However, this was not demonstrated in our study. On the contrary, the QTc prolongation was similar throughout the second dosing interval. Therefore, the timing of an ECG recording within the dosing interval seems irrelevant for chloroquine.

Furthermore, this study demonstrates that 19 patients unnecessarily had their treatment prematurely discontinued or had their dose adjusted due to a prolonged QTc interval based on the computerised interpretation of the ECG. In our study, electronically measured QTc values might differ from the manually performed measurement due to differences in standard lead selection, U‑wave recognition, U wave inclusion or exclusion, and definition of T‑wave ending [11, 13]. Another study found only a minor difference between the computerised and manual interpretation of the QTc interval [9]. However, the reliability of the computerised measurement of the QTc interval has been found to be questionable and manual measurement of the QTc interval is recommended [11, 13, 14]. Based on the present study, it is recommended that a cardiologist is consulted before clinical decisions are made based on the computerised interpretation.

A limitation of this study is the retrospective nature, although our large sample size included various ECGs captured at different time-points during the treatment with chloroquine. A possible bias may have been introduced by not manually recalculating all QTc intervals. However, computerised interpretation is commonly used in clinical practice; thus our study is a good representation of the normal clinical setting.

## Conclusion

Chloroquine treatment in COVID-19 patients gradually increased the QTc interval during the treatment period, most likely due to the pharmacokinetic profile of chloroquine. Due to a significant number of overestimated QTc intervals by computer analysis, it is advisable to measure the QTc interval manually before adjusting the dose or withdrawing this potentially beneficial medication.
